# Extracellular vesicles from *Trypanosoma cruzi*-dendritic cell interaction show modulatory properties and confer resistance to lethal infection as a cell-free based therapy strategy

**DOI:** 10.3389/fcimb.2022.980817

**Published:** 2022-11-16

**Authors:** Brenda Celeste Gutierrez, Maria Eugenia Ancarola, Izadora Volpato-Rossi, Antonio Marcilla, Marcel Ivan Ramirez, Mara Cecilia Rosenzvit, Marcela Cucher, Carolina Verónica Poncini

**Affiliations:** ^1^ Instituto de Investigaciones en Microbiología y Parasitología Médicas (IMPaM), Consejo Nacional de Investigaciones Científicas y Técnicas (CONICET), Buenos Aires, Argentina; ^2^ Programa de Pós-graduação em Biologia Celular e Molecular, Universidade Federal do Paraná, Curitiba, Paraná, Brazil; ^3^ Instituto Carlos Chagas - Fiocruz Paraná, Curitiba, Paraná, Brazil; ^4^ Departamento de Farmacia y Tecnología Farmacéutica y Parasitología, Universitat de Valencia, Valencia, Spain; ^5^ Departamento de Microbiología, Facultad de Medicina, Universidad de Buenos Aires (UBA), Buenos Aires, Argentina

**Keywords:** *T. cruzi*, dendritic cells, extracellular vesicles (EVs), cell free therapy, immunotherapy, dendric cells (DCs)

## Abstract

Extracellular vesicles (EVs) include a heterogeneous group of particles. Microvesicles, apoptotic bodies and exosomes are the most characterized vesicles. They can be distinguished by their size, morphology, origin and molecular composition. To date, increasing studies demonstrate that EVs mediate intercellular communication. EVs reach considerable interest in the scientific community due to their role in diverse processes including antigen-presentation, stimulation of anti-tumoral immune responses, tolerogenic or inflammatory effects. In pathogens, EV shedding is well described in fungi, bacteria, protozoan and helminths parasites. For *Trypanosoma cruzi* EV liberation and protein composition was previously described. Dendritic cells (DCs), among other cells, are key players promoting the immune response against pathogens and also maintaining self-tolerance. In previous reports we have demonstrate that *T. cruzi* downregulates DCs immunogenicity *in vitro* and *in vivo*. Here we analyze EVs from the *in vitro* interaction between blood circulating trypomastigotes (Tp) and bone-marrow-derived DCs. We found that Tp incremented the number and the size of EVs in cultures with DCs. EVs displayed some exosome markers and intracellular RNA. Protein analysis demonstrated that the parasite changes the DC protein-EV profile. We observed that EVs from the interaction of Tp-DCs were easily captured by unstimulated-DCs in comparison with EVs from DCs cultured without the parasite, and also modified the activation status of LPS-stimulated DCs. Noteworthy, we found protection in animals treated with EVs-DCs+Tp and challenged with *T. cruzi* lethal infection. Our goal is to go deep into the molecular characterization of EVs from the DCs-Tp interaction, in order to identify mediators for therapeutic purposes.

## Introduction

Extracellular vesicles (EVs) are a heterogeneous group of particles that includes among others three major groups according to their subcellular origin and size: apoptotic bodies, exosomes and microvesicles (Gurunathan et al., 2019; Bazzan et al., 2021). To date, increasing studies confirm that EVs mediate intercellular communication (Colombo et al., 2014). They are detected in most body fluids such as nasal secretion, feces, urine, blood and breast milk (Keller et al., 2006; Yáñez-Mó et al., 2015). Circulating EVs are high in patients with acute or chronic inflammation, preeclampsia, atherosclerosis, diabetes mellitus or cancer among other pathogenic conditions (Yamamoto et al., 2016). An important breakthrough was the discovery of nucleic acids in EVs such as mRNA and miRNA. RNA molecules cargo in EVs can be a selective process and several studies have shown that EV-associated mRNAs and miRNAs can be functionally transferred to recipient cells (Valadi et al., 2007; Baj-Krzyworzeka et al., 2016).

In pathogens, EV shedding is well described in fungi, bacteria, protozoan and helminth parasites (Ramirez and Marcilla, 2021; Wang et al., 2022). Of note, they were described mediating host-parasite interactions (Silverman et al., 2010a; Buck et al., 2014). In models of infection with *Leishmania* spp., it was described the importance of EVs in cell communication, the modulation of the immune response (Silverman et al., 2010a) and the importance of parasite glycoproteins regulating these processes, including conditioning antigen presenting cells (APCs) (Silverman et al., 2010b).

EV liberation and also their protein composition were previously described for *Trypanosoma cruzi* (da Silveira et al., 1979; Alves and Colli, 2008; Bayer-Santos et al., 2013; Cortes-Serra et al., 2022). Noteworthy, some studies have demonstrated the presence of mediators inside the EVs from *T. cruzi* regulating cellular functions in the host, adhesion and cell invasion (Gazos-Lopes et al., 2014; Neves et al., 2014; Wang et al., 2022), and new evidence suggests the EVs role in remote signaling and inflammation (Dantas-Pereira et al., 2021). Although *T. cruzi* does not possess the miRNA synthesis machinery, other small RNAs were found including transfer RNA-derived small RNA (stRNA), which make up the repertoire of small regulatory RNAs and are capable of modifying the genetic expression of host cells (Bayer Santos et al., 2013; Garcia Silva et al., 2014). The secretion of these EVs by *T. cruzi* would constitute a true information transfer mechanism between organisms from different kingdoms (Fernandez-Calero et al., 2015). In *T. cruzi* EVs there are glycoproteins from the trans-sialidase/gp85 superfamily (Ouaissi et al., 1990), and proteomic studies show the presence of α-Gal-glycoproteins, proteases and mucins normally membrane-associated by glycosylphosphatidylinositol or GPI anchors (Nakayasu et al., 2009; Torrecilhas et al., 2012; Bayer-Santos et al., 2013). Interestingly, animals injected with EVs from trypomastigotes (Tp) containing α-Gal residues increased amastigote nests in heart sections, triggered inflammation, and severe cardiac pathology (Trocoli-Torrecilhas et al., 2009). More recently, Lovo-Martins and colleagues have shown that the inoculation of EVs from Y *T. cruzi* strain prior to the infection reduces the inflammatory mediators and favors parasitism. *In vitro*, bone-marrow derived macrophages stimulated with these EVs before the interaction with the parasite increase its internalization and downregulate prostaglandin E2 and proinflammatory cytokines, suggesting a regulatory role for EVs supporting the parasite persistence (Lovo-Martins et al., 2018).

Immune cells are important targets for EVs and several reports demonstrate immune modulation by *T. cruzi* EVs (Torrecilhas et al., 2020; Dantas-Pereira et al., 2021). The parasite pathogen associated molecular patterns (PAMPs) are poorly detected at the initial steps of the infection (de Pablos Torró et al., 2018); however, *T. cruzi* EVs can trigger inflammatory responses and promote infection *via* TLR2 signalling in macrophages (Nogueira et al., 2015; Cronemberger-Andrade et al., 2020). EVs purified from peripheral blood of patients with Chagas induced proinflammatory cytokines in THP-1 cells (Groot-Kormelink et al., 2018), and both *T. cruzi* derived EVs from immune or non-immune cells are inflammatory for macrophages *in vitro*. The sensing of oxidized DNA inside EVs is involved in the activation of the inflammatory response *via* TLR9 and cGAS-PARP1 signalling pathways (Choudhuri and Garg, 2020).

Dendritic cells (DCs) are professional APCs, key players in prompting the immune response against pathogens and in self-tolerance maintenance, with a pivotal role capturing, processing and presenting antigens to T cells (Merad et al., 2013). Exosomes are considered immune regulators in DC-based immunotherapy (Markov et al., 2019). It is well described that DC-derived EVs can carry functionally active molecules on the surface such as complexes of MHC class I and II with antigens or costimulatory molecules (Munich et al., 2012). During the last decades, the use of EVs was also described such as an alternative immunotherapy approach in cancer (Yang et al., 2021) and numerous infections. EVs can interact with target cells and modify cellular activity by delivering different mediators. EVs can be presented as conventional carriers for RNAs, lipids and proteins, and appear as an alternative cell-free vectors for antigen delivery. Tumor-derived EVs stimulate antitumor immunogenicity in DCs (André et al., 2002) and more interestingly, Ag-pulsed DC derived exosomes display prophylactic and therapeutic properties in tumour-bearing mice (Zitvogel et al., 1998). Immune protective response triggered by EVs from Ag-loaded DCs has also been described for different pathogens such as *Toxoplasma gondii* (Aline et al., 2004; Jung et al., 2020), *Eimeria* spp. (del Cacho et al., 2012) and *Leishmania major* (Schnitzer et al., 2010). However, no previous results in the field were described for *T. cruzi*.

Our group and others have previously demonstrated that *T. cruzi* downregulates DCs immunogenicity *in vitro* and *in vivo* (2015; Poncini et al., 2008; Gil-Jaramillo et al., 2016). Here we analyze EVs from the *in vitro* interaction between blood circulating Tp and bone-marrow derived DCs. We found that Tp increases the number and size of EVs in cultures with DCs. EVs displayed some exosome markers, and intracellular RNA. By proteomics we found that the presence of the parasite in DCs cultures has an impact in the protein composition of EVs. In addition, EVs from the interaction of Tp-DCs are easily uptaken by unstimulated DCs and modify the activation status of LPS-stimulated DCs. Finally, we found that the prophylactic treatment with EVs from DCs co-cultured with Tp (EVs-DCs+Tp) partially protects animals from the lethal infection, proposing EVs as promising mediators for therapeutic purposes against Chagas disease.

## Materials and methods

### Animals and parasites

Eight-to-ten week old C3H/HeN, C57BL/6 and CF1 male mice were obtained from the animal facilities of IMPaM UBA-CONICET, School of Medicine, University of Buenos Aires. Animals were bred under sanitary barrier in specific-pathogen-free conditions.

Parasites from RA strain (González et al., 1981) were maintained by weekly intraperitoneal inoculation of three weeks-old male CF1 mice (1 × 10^5^ parasites/mouse). RA bloodstream forms (Tp) were obtained from whole blood at the peak of parasitemia 7 days post-infection (dpi), thoroughly washed and purified by density gradient centrifugation as previously reported (Poncini et al., 2008). For the lethal infection challenge, ten-to-twelve week old C57BL/6 male mice received intradermic (hindfoot) injection with 1000 parasites as previously described (Poncini et al., 2015; Poncini et al., 2017; Gutierrez et al., 2021). Animal health condition, parasite load and mortality were periodically recorded.

All experiments were performed according to protocols CD N° 04/2015 approved by the University of Buenos Aires´s Institutional Committee for the Care and Use of Laboratory Animals (CICUAL) in accordance with the Council for International Organizations of Medical Sciences (CIOMS) and International Council for Laboratory Animal Science (ICLAS) international ethical guidelines for biomedical research involving animals.

### Cell culture and EV isolation

DCs were differentiated as previously described (Poncini et al., 2008). Briefly, femurs and tibias from C3H 8-12 week-old mice were flushed and bone marrow cells were incubated for 7 d in IMDM complete medium supplemented with: 10% (v/v) heat-inactivated FCS (Internegocios, Argentina), penicillin (100 U/mL) and streptomycin (100 mg/mL), 2-mercaptoethanol (50 µM); with 20% conditioned medium from GM-CSF-producing J558 cells. After 7 days, cells were harvested, washed, plated (1 × 10^6^ cells/mL) and cultured in serum free medium with or without Tp (1:2 cell: parasite) for 20 h. Control DCs were cultured with medium alone. Activated DCs were treated with a low dose of LPS (50ng/mL). Cell viability was assessed before and after culture by Trypan blue staining at 0.2% final concentration. Cell viability for experiments was 85% or more.

For EVs isolation, culture supernatants were collected and subjected to successive centrifugation steps according to Théry et al. (2006). At least 5 mL of culture medium was centrifuged at 300 × g for 10 min in order to pellet cells, and then supernatants were harvested and centrifuged at 2,000 × g for 20 min and at 10,000 x g for 30 min. Finally, supernatants were ultracentrifuged at 100,000 × g for 70 min at 4°C (Beckman Coulter Optima L-100 XP centrifuge using a fix angle rotor). Pellets were washed with PBS and ultracentrifuged at 100,000 × g for 70 min at 4°C and then resuspended in PBS and used for transmission electron microscopy (TEM), RNA isolation, protein characterization and functional studies.

### Transmission electron microscopy (TEM)

Cellular pellets and EVs resuspended in PBS were fixed in Karnovsky’s fixative (0.5% glutaraldehyde, 2.5% paraformaldehyde), and processed according to Marcilla et al. (2012) at the Service of Microscopy, Servicios Centrales de Soporte a la Investigación Experimental (SCSIE), Universitat de València, Spain and analyzed by TEM.

### Nanoparticle tracking analysis

For Nanoparticle Tracking Analysis (NTA), each pellet was suspended in filtered PBS (1:50) and analyzed with a Nanosight LM10 (Malvern™, U.K.). Readings were performed in triplicate during 60 sec videos at 10 frames per sec at room temperature, with the following parameters: camera level 9, screen gain 9, detection threshold 6. The mode size and the concentration of particles resulting from 2 independent replicates from 2 pooled samples for each treatment.

### Flow cytometry

**Figure 1:**
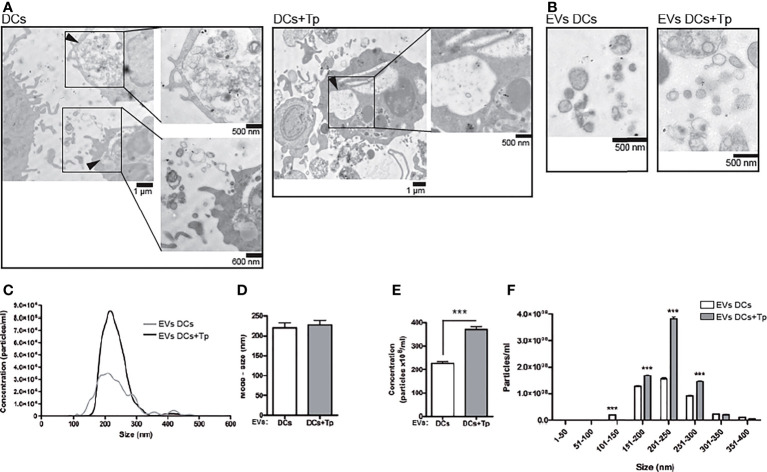
EVs from DCs+Tp presented higher diameters and abundance than EVs DCs. **(A)** Multivesicular compartments in DCs (upper panel) and DCs+Tp (lower panel) analyzed by TEM. Black arrows show amplified images. Bars, 500nm, 600 nm and 1µm. **(B)** EVs were isolated after sequential centrifugation from cultures of DCs at 1 ×10^6^ cells/mL and 1:2 relation DC : Tp and processed for TEM. Bar, 500 nm. **(C)** Concentration distribution profile of EVs by size from 0 to 600 nm by Nanoparticle Tracking Analysis. **(D)** Mode size of EVs derived from DCs or DCs+Tp. **(E)** Concentration of EVs based in size ranges. Statistical analysis was performed with Wilcoxon test, ***p<0.001. **(F)** EVs size distribution in 50 nm ranges. Statistical analysis was performed with two-way ANOVA and Bonferroni´s post-test, ***p<0.001.

For EVs characterization, samples were stained for 15 min at 4°C with anti-MHCII (FITC, M5/114.15.2) and anti-CD9 (biotin, MZ3) all from Miltenyi Biotec. The secondary reagent was Cy5-streptavidin (BD Biosciences). After washing in filtered PBS EV suspensions were diluted to a protein concentration of 5 µg/mL and analyzed in a CytoFLEX LX flow cytometer (Beckman Coulter). Particle detection was calibrated by using CytoFLEX fluorospheres from 160 to 900 nm (Beckman Coulter), as observed in **Figure 1D** (right panel). Acquisition was performed at continuous flow yielding the events per 30 µL in order to calculate EVs concentration per sample.

For T cell characterization, single cell suspensions were incubated with the following fluorophore-conjugated anti mouse monoclonal antibodies (Ab) for 30 min at 4°C: anti-CD3 (biotin, 145-2C11), anti-CD4 (FITC, GK1.5), anti-CD8 (PerCP, 53-6.7) all from Miltenyi Biotec. PE-Cy7-streptavidin was used as secondary regent (BD Biosciences).

For intracellular staining, cells were incubated with *T. cruzi* Ag at 20 µg/mL and brefeldin A (10 µg/mL; Sigma) for 5 h as previously described (Poncini et al., 2015). After surface staining cells were washed, fixed and permeabilized with Cytofix/Cytoperm solutions (BD Biosciences) and stained with anti-INF-γ (PE, W18272D; BioLegend). Cells were acquired in a FACSAria flow cytometer and analyzed using FlowJo 7.6. Gating strategy to study T cells is depicted in **Supplementary Figure 4**.

### RNA isolation, protein purification and characterization

RNA from EVs was isolated with Trizol LS (Life Technologies) according to Ancarola et al. (2017). RNA integrity was analyzed by gel electrophoresis and the concentration was determined using a Qubit Fluorometer (Invitrogen).

To confirm the intravesicular location of the isolated RNA, samples were differentially treated or not, prior to RNA isolation as follow: proteinase K (0,5 μg/μL) 10 min at 37°C, 10 min at 65°C followed by incubation with RNAse A (0.04 μg/μL), depending on the sample and as it was described by Ancarola et al. (2017). The corresponding RNA profiles were analyzed by capillary electrophoresis in Fragment Analyser (Advanced Analytical Technologies, U.S.A.).

For protein analysis, immunoblot or liquid chromatography coupled with tandem mass spectrometry (LC-MS/MS) was performed on ultracentrifugation pellets according to Marcilla et al. (2012). For immunoblots, approximately 15 µg of cellular protein determined by Bradford (1 ×10^6^ DCs) or the EVs-pellet obtained from 5 × 10^6^ cells (cultured at 1 ×10^6^ cells/mL) were resuspended in ice-cold lysis buffer (20 mM Tris-acetate, pH 7.0, 1 mM EGTA, 1% Triton X-100, 0.1 mM sodium fluoride, 5 μg/mL leupeptin, 1 mM sodium orthovanadate, 1 mM phenylmethylsulfonyl fluoride). Proteins were resolved by SDS-PAGE, transferred to nitrocellulose membranes (Amersham) and transfer verified by reversible membrane staining with Ponceau Red (5% w/v) in 1% (v/v) acetic acid. Then, membranes were probed with anti-CD63 polyclonal Ab (SC-31211, Santa Cruz), anti-MHCI monoclonal Ab (Sc-59309, Santa Cruz), anti-CD9 monoclonal Ab (MZ3, Miltenyi Biotec) and HRP-conjugated secondary Ab. Detection was assayed using ECL chemiluminescent system (Amersham) according to manufacturer’s instructions. Three independent samples of EVs from DCs or DCs+Tp were analyzed.

### Proteomics and bioinformatics analysis

Liquid chromatography coupled with tandem mass spectrometry (LC-MS/MS) was performed on EVs ultracentrifugation pellets according to Marcilla et al. (2012). The proteomic analysis was executed at the proteomics facility of SCSIE University of Valencia that belongs to ProteoRed, PRB2‐ISCIII, and is supported by grant PT13/0001, of the PE I+D+i 2013‐2016, funded by ISCIII and FEDER. The Paragon algorithm of ProteinPilot v 4.5 was used to search the NCBI complete database with the following parameters: trypsin specificity, cys‐alkylation, no taxonomy restriction, and the search effort set to through. Reported results correspond to those proteins showing unused score ≥ 1.3 (identified with confidence ≥ 96%), ≥ 2 distinct peptides having at least 95% confidence and *T. cruzi* or mouse protein sequence annotation. Proteins annotated under the terms “hypothetical protein”, “expressed protein” or “conserved protein” were searched for domains in the domains database CDART at the NCBI site, and re-annotated if necessary, as previously described (Ancarola et al., 2017).

For protein analysis, a Venn diagram was generated using the online tool Venny 2.1 (Oliveros, 2007). PANTHER database was used to retrieve the gene ontology (GO) terms for molecular function and cellular components of EV proteins from DCs and DCs+Tp. GO term descriptions were then downloaded from The European Bioinformatics Institute site (https://www.ebi.ac.uk/QuickGO/) as previously described (Ancarola et al., 2017).

### Labelling of EVs and functional assay

DCs were labelled with the lipophilic dye PKH26 (Sigma-Aldrich) according to manufacturer’s protocol, washed, and then cultured with or without Tp. DCs-derived EVs were enriched as described above. PKH26^+^ DC-derived EVs were then isolated and used for functional assays. EVs isolated from the culture of 2.5 × 10^6^ cells DCs or DCs+Tp were added to DCs cultures (5 × 10^5^ cells per well) for 24 h. Internalization by DCs was identified by confocal microscopy and flow cytometry. In addition, DC activation status was determined by flow cytometry as described below.

### Determination of EVs internalization and DCs activation by confocal microscopy and flow cytometry

For confocal microscopy, cells were harvested, washed and fixed in cold methanol for 10 min and DAPI (Invitrogen™) staining was used for nuclear visualization on an Olympus FV1000 microscope (lasers exciting at 405 and 559 nm, × 60 objective) using Olympus Fluoview (version 4.2b) at the confocal microscopy service of Instituto de Fisiología y Biofísica (IFIBIO-Houssay), School of Medicine, UBA. Images were analyzed using the ImageJ software (version 1.52p).

For flow cytometry and depending on the assay, the following Ab were used: anti-MHCII (FITC, M5/114.15.2), and anti-CD11c (Biotin, N418) all from Miltenyi Biotec. The secondary reagent was Cy5-streptavidin (BD Biosciences).

For DCs surface staining, cellular suspensions were incubated with fluorescent-labeled Ab, 30 min at 4°C. Sample acquisition was achieved on FACSAria flow cytometer (BD Biosciences) and analyzed by FlowJo 7.6 software.

### ELISA

After 20 h of culture, cell supernatants were harvested and stored at -80°C until used. Mouse IL-10 or TNF-α was detected by ELISA (R&D Systems, Minneapolis, MN) according to manufacturer’s protocol.

### Experimental treatment with EVs and analysis of the effector response after the infection challenge

EVs from DCs or DCs+Tp were obtained as described above. PBS or EVs generated from 1 × 10^6^ cells were intradermically injected per mice at day 0. At day 7 post EVs or PBS injection, animals were challenge with the lethal infection. To this end, ten-to-twelve week old C57BL/6 male mice received intradermic (id, hindfoot) injection with 1000 RA parasites (Gutierrez et al., 2021). Experimental procedure included four to five animals per group depending on the experiment and was defined as: i) PBS, negative for infection; ii) PBS+Tp, positive for infection; iii) EVs DCs treatment+Tp; iv) EVs DCs+Tp treatment+Tp. Animal health condition, parasite load and mortality were periodically recorded.

In a second round of experiments a second immunization dose with EVs was applied at day 7 after the first one, followed by the challenge with the parasite 10 days after the treatment. Immune response was studied at day 20 pi. To this end, T cell populations and intracellular INF-γ production were analyzed in cell suspensions from popliteal lymph nodes (pLN) by flow cytometry, as described above. Cell suspensions were obtained after mechanical disaggregation of pLNs in a 100 µm nylon mesh as previously described (Poncini and González-Cappa, 2017). Cell viability was assessed by Trypan blue dye exclusion. Experimental procedure included four to five animals per group depending on the experiment, and two to three repetitions.

### Statistical analysis

Student´s *t*-test or non-parametric Wilcoxon tests were performed in order to analyse statistical significance between two samples. For more samples ANOVA and Dunnett´s or Bonferroni´s post-test or the non-parametric Mann-Whitney U test were applied. Survival curves were analyzed by Kaplan-Meier. Analyses were carried out with GraphPad Prism 4 software for Windows. P<0.05 value was defined as significant.

## Results

### Identification and quantification of EVs from the interaction of DCs with T. cruzi *in vitro*

Previous studies demonstrated the capacity of DCs to secrete EVs to the extracellular milieu (Zitvogel et al., 1998); especially four different populations of DC-EVs were described with variable size and protein content (Kowal et al., 2016). Particularly, the maturation state of these cells can influence the subcellular protein distribution and also the EVs-DCs composition and size (Théry et al., 2009; Ten Broeke et al., 2011). Ultracentrifugation and density gradients are still the most common methods for EV purification and EV structural characterization is currently carried out by transmission electron microscopy (TEM) (Royo et al., 2020).

We found by TEM that both control and Tp co-cultured DCs (DCs+Tp) presented structures compatible with internal multivesicular compartments next to the plasmatic membrane (**Figure 1A**, black arrows).

Here, small EVs were isolated from culture supernatants after differential ultracentrifugation (**Figure 1B**). Pellets obtained after 300, 2,000 and 10,000 x *g* centrifugation were discarded and only small EVs from 100,000 x *g* were analyzed. Size distribution determined by TEM showed a heterogeneous population of vesicles with diameters ranging from 30 to 200 nm in DCs cultures and from 60 to 400 nm in samples from DCs co-cultured with Tp (**Supplementary Figure 1A**). Particles derived from Tp-DC co-cultures (EVs DCs+Tp) were more abundant, as randomly quantified across 20 fields, and showed larger diameters than EVs DCs (**Supplementary Figure 1B**). The evaluation by Nanoparticle Tracking Analysis (NTA) effectively demonstrated that Tp induced a significant increase in EV release to milieu by DCs (**Figures 1C, E**). A similar mode for the size of EVs DCs and EVs DCs+Tp populations was observed (**Figure 1D**); however the EVs size profile distribution shows a higher proportion of EVs in the range from 150 to 300 nm for EVs DCs+Tp, consistent with microvesicles/ectosomes and confirming the TEM findings (**Figure 1F**).

### Different RNA and protein content in EVs DCs+Tp compared with EVs DCs

The analysis of the RNA and the protein cargo showed that EVs DCs contain almost exclusively small RNA (<200 nt) (**Supplementary Figure 2A**, panel a). On the contrary, EVs DCs+Tp showed a different RNA pattern including RNA species around 200 and 500 nt (**Supplementary Figure 2A**, panel b). This pattern also differed from the corresponding to ribosomal 18S and 28S RNAs found in cell samples (**Supplementary Figure 2B, C**). To avoid possible contamination with `free´ RNA co-sedimented with EVs in the ultracentrifugation step and to confirm that the results obtained corresponded to intravesicular RNA, isolated EVs were treated with proteinase K and RNase A and then analyzed. RNA patterns from DCs and DCs+Tp EVs treated or not-treated were comparable, confirming the intravesicular location of the RNA found (**Supplementary Figure 2A**; a versus c, and b versus d).

**Figure 2:**
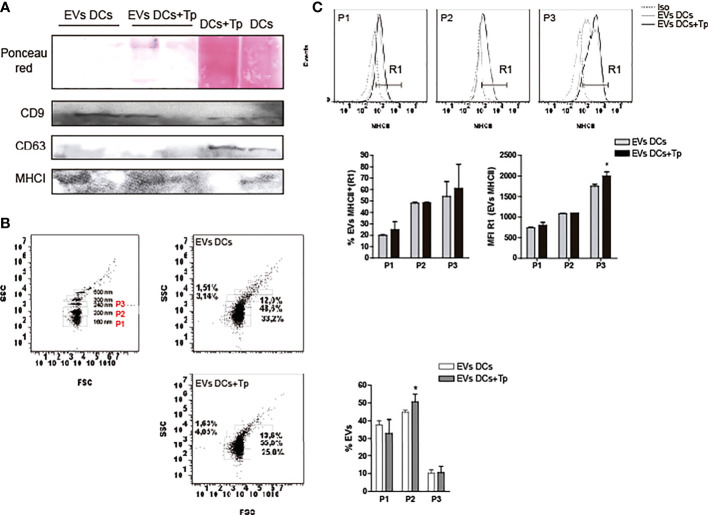
EVs protein characterization. **(A)** Detection of CD9, CD63 and MHCI in EVs DCs, EVs DCs+Tp and total cell lysates by immunoblot. Ponceau red staining confirmed the correct protein transfer to membranes prior to Ab incubation. B-C) EVs analysis by flow cytometry. **(B)** Gating strategy was performed based on the known sizes of commercial beads (left panel). Based in EVs concentration detected in samples, three major subpopulations were defined in EVs DCs and EVs DCs+Tp (P1, P2 and P3; right panels). **(C)** Percentage and median fluorescence intensity of the MHCII marker was analyzed in MHCII^+^ EVs (R1) in P1, P2 and P3. Data are representative of at least two biological replicates for each treatment. For comparisons between two groups student’s t-test was used. *p < 0.05.

Previous reports demonstrate that not only plasmatic membrane-derived vesicles from DCs, but also endosome-derived membrane vesicles can bear MHC class I and II molecules (Zitvogel et al., 1998; Kowal et al., 2016) and co-stimulatory molecules, features that enhance their immunotherapeutic potential as a cell-free strategy (Markov et al., 2019). In addition, the presence of tetraspanins such as CD9, CD63 and CD81 propose, to some extent, a protein signature for DCs exosomes (Kowal and Tkach, 2019). Apparently the composition of exosomes in DCs is related to cell maturation state (Segura et al., 2005). Interestingly, tetraspanins have been reported to be involved in antigen presentation by MHC interaction and by the immune synapse formation in DCs or other APCs (Unternaehrer et al., 2007; Petersen et al., 2011). Here, we observed by western blot that EVs DCs and EVs DCs+Tp contain CD9 and MHC class I molecules, also detected with different intensities in total cell lysates (**Figure 2A**). No CD63 was detected in EVs, although CD63 signal was clearly observed in total cell lysates (**Figure 2A**). By flow cytometry, we also confirmed that EVs DCs+Tp displayed an enlarged population from 200-230 nm (P2) compared to EVs DCs (**Figure 2B**) as observed by TEM and NTA. Furthermore, we found a variable amount of MHCII in different subpopulations of EVs (P1, P2 and P3), both in EVs DCs and EVs DCs+Tp samples (**Figure 2C**). P2 and P3 subpopulation showed higher expression of MHCII. Interestingly, P3 in EVs DCs+Tp displayed increased median fluorescence intensity (MFI) of MHCII compared to EVs DCs (**Figure 2C**).To identify the protein content in EVs DCs and EVs DCs+Tp enriched fractions we performed an exploratory analysis by liquid chromatography coupled with tandem mass spectrometry (LC-MS/MS). We observed that both DCs and DCs+Tp EVs contained the presence of proteins typically found in exosomes derived from DCs such as CD9, MHCII, annexins, GAPDH and enolase (**Supplementary Figure 3A**), as previously reported (Kowal et al., 2016). However, some proteins were differentially detected in EVs from DCs versus DCs+Tp as expressed in the Venn diagram and GO terms for molecular function and cellular component (**Supplementary Figures 3A, B**, respectively). Interestingly, other proteins were detected with a lower unused score or number of peptides such as CD81, Alix, HSP70, Syntenin-1, TSG101, Galectin-1/3 and *T. cruzi* trans-sialidase, the last one only in EVs DCs+Tp (The mass spectrometry proteomics data have been deposited to the ProteomeXchange Consortium via the PRIDE (Perez-Riverol et al., 2022) partner repository with the dataset identifier PXD037795.).

### EVs from T. cruzi-DCs interaction modulate DC activation *in vitro*

**Figure 3:**
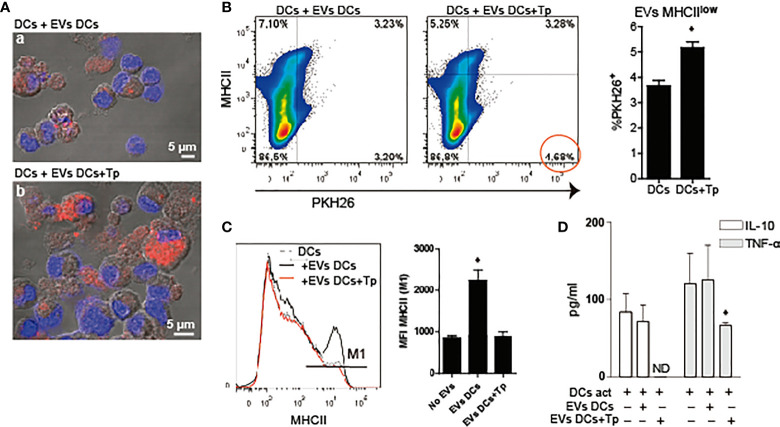
DCs-derived EVs are internalized by DCs in culture. **(A)** Confocal microscopy of DCs incubated with PKH26^+^ EVs (red) derived from DCs (a) or DCs+Tp (b) for 24 h at 37°C. Scale indicates 5 µM. **(B)** Analysis of MHCII expression associated to PKH26^+^ EVs uptake by DCs in culture by flow cytometry. Statistical analysis was performed with Student´s *t*-test, *p<0.05. **(C)** Analysis of MHCII median fluorescence intensity (MFI) in DCs stimulated or not with EVs-DCs or EVs-DCs+Tp. Statistical analysis was performed with ANOVA and Dunnett´s test, *p<0.05. **(D)** TNF-α and IL-10 in culture supernatants detected by ELISA. Statistical analysis was performed with ANOVA and Dunnett´s test, *p<0.05. Data are representative of at least three biological replicates for each treatment.

Previously, we have reported that Tp interaction confers tolerogenic properties to DCs *in vitro*. However, this effect was observed with the whole parasite and not with medium enriched in Tp secretion products (Poncini et al., 2008). Here we studied the effect of DCs-derived EVs and EVs product of the parasite-DCs interaction over DCs in a steady and/or activated state. To this end, first we analyzed if EVs can be uptaken by DCs. EVs labeled with the lipid dye PKH26, were incubated with DCs in steady-state and uptake was determined by confocal microscopy and flow cytometry. By confocal microscopy we confirmed that both types of EVs were uptaken by DCs. While not quantified by microscopy, DCs incubated with EVs-DCs+Tp showed cells with high PKH26 incorporation, compatible with high EV uptake (**Figure 3A**, a versus b). By flow cytometry, we confirmed that DCs with MHCII^int/low^ expression incorporate more EVs DCs+Tp than EVs DCs (**Figure 3B**). In addition, DCs incubated with EVs DCs slightly increased MHCII expression, while the EVs-DCs+Tp treated displayed the same expression observed in control DCs and expressed as mean fluorescence intensity (MFI) (**Figure 3C**). Next, we analyzed by ELISA the effect of EVs on TNF-α and IL-10 secretion in DCs activated with LPS. We found no differences in cytokine secretion between control DCs and DCs incubated with EVs DCs (**Figure 3D**). Interestingly, EVs DCs+Tp downregulated the IL-10 and TNF-α secretion, the former at undetectable levels (**Figure 3D**). Of note, no differences in cell viability were detected in cultures (data not shown). These results suggest that EVs from DCs+Tp modulate to some extent MHCII expression in DCs, also IL-10 and TNF-α secretion to the milieu.

### EVs derived from the interaction between DCs and Tp partially protect mice from lethal infection

EVs derived from DCs have lately become an attractive tool since they can cargo specific antigen and display immunogenicity. A cell-free-based vaccine using EVs has been previously described (Keller et al., 2006). In addition, DCs derived EVs were reported to induce protective immunity in *Toxoplasma gondii* (Aline et al., 2004), *Leishmania major* (Schnitzer et al., 2010), among other infections. However, while there are extensive studies on EVs derived from *T. cruzi* (Dantas-Pereira et al., 2021), and their role in the host immune regulation (D’Avila et al., 2021), there is little information about therapy approaches with DCs in experimental Chagas disease.

**Figure 4:**
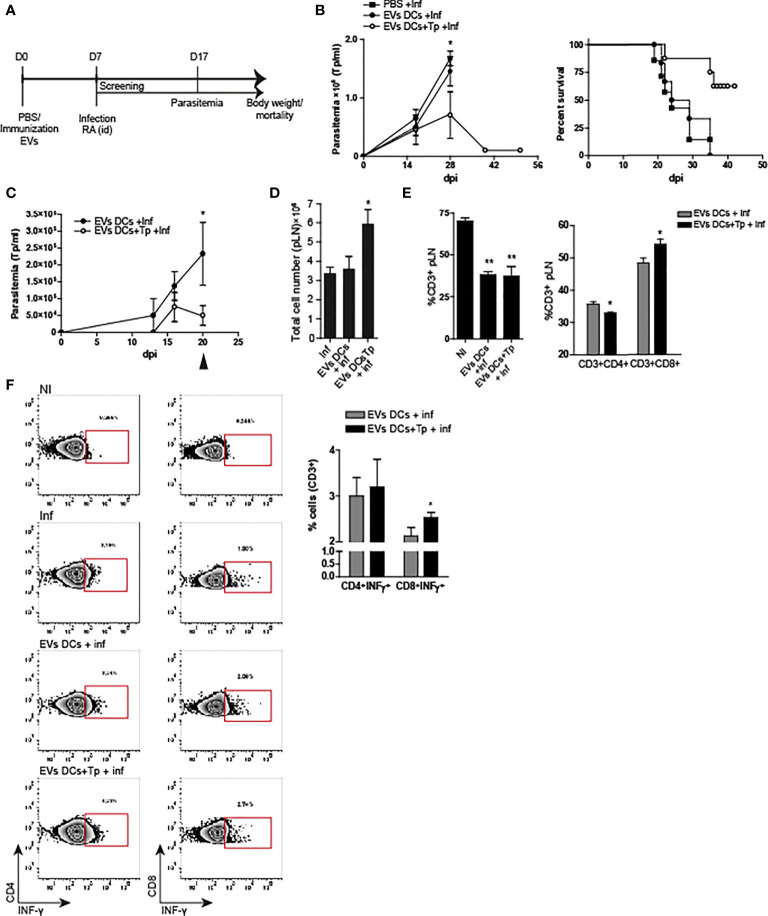
EVs DCs+Tp protect mice from lethal *T. cruzi* infection. **(A)** Schematic representation of the experimental design. **(B)** Peripheral blood parasitemia and mice survival. Animals were monitored during experimental infection. Control of infection (squares), EVs DCs treated and challenged (filled circle) and EVs DCs+Tp treated and challenged (fill circle) as described in materials and methods. **(C)** Peripheral blood parasitemia in animals treated with a second dose of EVs 7 days after the first application and challenged with RA 10 days after the treatment. **(D)** Absolute cell number in popliteal lymph nodes (pLN) 20 days post-infection. **(E)** Percentage of CD3^+^ T cells in pLN (left panel) and percentage of CD4^+^ and CD8^+^ T cells in pLN (right panel) by flow cytometry. No differences were detected between no-treated infected (Inf) and treated animals (data not shown). **(F)** Intracellular INF-γ detection from ag-specific CD4^+^ or CD8^+^ T cells by flow cytometry. One representative assay of the gating strategy for INF-γ detection in CD4^+^ and CD8^+^ T cells is shown (left panels). Bar graph representation summing up the values obtained from the biological replicates in independent experiments (right panel). One of three representative experiments designed with 4-5 mice per group is shown. For more than two treatments, statistical analysis was performed with ANOVA and Dunnett´s test. For comparisons between two groups student´s t-test was used. *p< 0.05, **p<0.01.

Here we analyzed the effect of one dose of EVs-DCs+Tp and EVs DCs injected i.d. as described in material and methods, 7 days before the challenge with the infection with the lethal RA strain (Poncini et al., 2015). Parasitemia, body weight and mortality were registered, and animals losing more than 25% of their body weight were euthanized (represented in **Figure 4A**). Treatment with EVs-DCs+Tp partially protected animals from the lethal infection; with approximately a 60% of survival (one representative experiment of three independent studies with similar results is shown; **Figure 4B** right panel). In addition, EVs-DCs+Tp treatment reduced the number of circulating parasites at the peak of parasitemia (28 dpi, **Figure 4B** left panel).

In order to analyze the immune response after the treatment in animals challenged with the lethal infection, we set a second round of experiments with a second immunization with EVs at day 7 after the first dose, followed by the challenge with the parasite 10 days after. Immune response was studied at 20 dpi. We found that animals treated with two EVs DCs+Tp doses controlled the parasitemia earlier in time in comparison with the ones that received one dose. In addition, treated animals, showed a marked decline in blood-Tp since 16 dpi (**Figure 4C**). No differences were observed between infected animals and EVs DCs treated and challenged (data not shown). We found a sharp adenomegaly in draining lymph node (pLNs), with high absolute cell-number in EVs DCs+Tp treated animals (**Figure 4D**). Although treatment with EVs DCs+Tp did not modify the proportion of total CD3^+^ T cells during the infection (**Figure 4E**, left panel; EVs DCs versus EVs DCs+Tp treated), EVs DCs+Tp treatment enhanced the percentage of CD8^+^T cells (**Figure 4E**, right panel). Of note, it was detected more INF-γ production by ag-specific CD8^+^ T cells (**Figure 4F**), compatible with a stronger effector T-cell response. These results provide the evidence that *T. cruzi*-DCs derived EVs are an attractive cell-free strategy for immunotherapy in Chagas disease.

## Discussion

It is reasonable to assume that in protozoan infections the intracellular stages are the responsible for the EV exchange between the parasite and host cells, as described for amastigotes in *Leishmania major* (Silverman and Reiner, 2012). However, a paracrine/juxtacrine cross-talk between Tp (the extracellular forms of the parasite) and host cells through EVs secretion was described by Ramirez et al. (2017). In addition, it was also demonstrated a Ca^2+^ dependent up-regulation in the secretion of membrane derived vesicles in THP-1 monocytes induced by Tp (Cestari et al., 2012); nevertheless, the precise mechanism and the signaling cascades stimulated by the parasite are still unknown. Interestingly, previous reports show that low pH and other stress signals increment the release of EVs by the parasite which reach a peak at 120 min at 37°C *in vitro* (Vasconcelos et al., 2021), suggesting its possible participation in parasite invasion.

On the other hand, not only parasite products but also EVs released by human peripheral blood mononuclear cells infected with SylvioX10/4 strain of *T. cruzi* activate THP-1 in culture. Interestingly, it was shown that in infected mice and chronic patients with Chagas disease most of the vesicles are of leukocyte or endothelial origin (Chowdhury et al., 2017; Choudhuri and Garg, 2020).

In the present work we found that *T. cruzi* is capable of modulating the secretion of EVs derived from DCs *in vitro*. Due to *T. cruzi*-DC interaction time in cultures, the effects described here involve the Tp stage of the parasite. During the first 24 h of culture, and depending on the multiplicity of infection, the percentage of cells infected with amastigotes can be low (Poncini et al., 2008). Results obtained by proteomics showed low *T. cruzi* protein cargo in EVs (only trans-sialidase was detected), suggesting that most of the EVs released in supernatants are from DC origin. However, the presence of Tp modifies the abundance and the size of the EVs shedding to milieu by DCs.

While exosome-like particles display diameters up to 100 nm, ectosomes or microvesicles whose origin is the plasma membrane, display among 100-350 nm (Cocucci and Meldolesi, 2015; Witwer and Théry, 2019). In addition to modify the number and size of the EVs released, here we found that Tp apparently, change the RNA and protein content in EVs from DCs. These results suggest that the parasite could shape the nature and probably the origin of the EVs released by DCs; however more studies are needed in order to confirm this affirmation.

DCs in different maturation status delivered exosomes with different RNA cargo, especially miRNAs. Interestingly, immature DCs secrete high quantity of exosomes (Montecalvo et al., 2012). Here we observed that EVs DCs+Tp in addition to small RNA, present populations of longer RNA (>180nt and >400nt), different from large ribosomal RNA. No previous reports were found about these RNA populations in EVs and *T.cruzi*. This result needs further studies since these RNAs could represent long non-coding RNAs or mRNA transcripts. Long non-coding RNAs are heterogeneous group of regulators with a length of more than 200 nt and their description in EVs has not been thoroughly studied enough (Wang et al., 2019).

In the same line, we found different protein content between DCs and DCs+Tp EVs. It was previously described that tetraspanins are differentially expressed by human and murine subsets, and they display a key role in DC receptors regulation, including antigen presentation (Zuidscherwoude et al., 2017). As expected for murine DCs, we found more CD9 than CD63 expression in cell lysates and in EVs by immunoblot and LC-MS/MS. Interestingly, in DCs and DCs+Tp EVs we also detected MHCII. Previous studies reported the association of CD9 and MHCII for the formation of multimers in the membrane of DCs and by this interaction an enhanced efficiency for antigen presentation (Unternaehrer et al., 2007). On the contrary, tetraspanins content in EVs can be variable and in the case of small EVs isolated by ultracentrifugation MHCII might be abundant both in CD9^+^ or CD9^-^ particles (Kowal et al., 2016). CD63 is an accepted marker found in exosomes and predominantly an intracellularly expressed tetraspanin. In addition it was observed to be involved in the internalization of complex ag (Mantegazza et al., 2004). Previous reports demonstrate that tetraspanins can be differentially expressed by different DCs subsets and CD9 is highly expressed in murine myeloid DCs (Zuidscherwoude et al., 2017). Here we found that EVs display CD9, while no CD63. Future studies would explain if this result is associated to the particles origin or if another mechanism is involved in the lack of CD63 in EVs.

By proteomics we observed that EVs DCs and EVs DCs+Tp share the presence of certain proteins; however, EVs DCs+Tp displayed an extended list of protein cargo. Of note, here we demonstrate that the larger population of EVs from DCs+Tp displays more MHCII. This result is extremely interesting since our group and others have previously described that Tp downregulate the surface expression of MHCII in DCs (Poncini et al., 2008). The release of MHCII molecules in EVs could be a possible evasion mechanism that the parasite exploits in order to modulate DCs immunogenicity. Studies in course may answer this question.

DC based vaccines have great potential for the treatment of different disorders, especially in cancer (Sprooten et al., 2019). Different approaches were described ranging from passive loading of *in vitro* differentiated DCs with tumor or pathogen peptides to transfection with nucleic acids or viral vector encoding diverse antigens. Although the FDA has approved monocyte-derived DC therapy for cancer, this presented some limitations (Palucka and Banchereau, 2013). One of the most important problems for DC-based vaccines, in addition to the high price, is the correct delivery of the antigen. In this context, EVs derived from DCs have lately become an attractive tool for cell-free-based vaccine since they display immunogenicity; can load specific antigens and the structure for antigen presentation (Markov et al., 2019). *In vitro* we found a greater uptake of EVs DCs+Tp than EVs DCs by control DCs. Surprisingly, EVs DCs+Tp modulated the activation status of DCs in culture. Not only MHCII surface expression, but also both IL-10 and TNF-α secretion were decreased. *In vivo* immunization with EVs as a cell-free immunotherapy shows that mice treated with only one dose of EVs-DCs+Tp have higher survival and low parasitemia after the challenge with the lethal infection with *T. cruzi*. Animals treated with two doses of EVs DCs+Tp showed fewer circulating parasites compared to those treated with one-dose and improved effector response of ag-specific T cell.

In conclusion, the results presented here, as well as go deep in the understanding of the parasite and the host interplay, suggest that *T. cruzi*-DCs derived EVs are a novel cell-free strategy as immunotherapy against Chagas disease. The results also propose EVs better molecular characterization for future development of new accessible synthetic tools.

## Data availability statement

The mass spectrometry proteomics data have been deposited to the ProteomeXchange Consortium via the PRIDE (Perez-Riverol et al., 2022) partner repository with the dataset identifier PXD037795.

## Ethics statement

The animal study was reviewed and approved by CICUAL CD N° 04/2015.

## Author contributions

BG, MA, MC, and CP contributed with the conception and the design of the study. BG and CP performed the experiments and analyzed data. MA and MC contributed with RNA purification and analysis of the results. AM contributed to TEM and LC/MS-MS. IR and MIR contributed with EVs quantification, the writing and the analysis of the results. CP wrote sections of the manuscript. All authors contributed to manuscript revision. All authors contributed to the article and approved the submitted version.

## Funding

This work was supported by Fundación Bunge & Born, Universidad de Buenos Aires (UBACyT 2017 20020160100117BA and 2020 20020190100230BA), Grant for Research on Infectious Diseases and Agencia Nacional de Promoción Científica y Tecnológica (ANPCyT), Argentina, projects PICT2017 N°2062. AM is supported by the Agencia Estatal de Investigación, Ministerio de Ciencia e Innovación, Spain (Grant number PID2019-105713GB-I00/AEI/10.13039/501100011033), and Conselleria d’Educació, Cultura i Esports, Generalitat Valenciana, Valencia, Spain (Grant PROMETEO/2020/071).

## Acknowledgments

We thank Eduardo Gimenez, Ricardo Chung and Marianela Lewicki for technical assistance. The Service of Proteomics, Servicios Centrales de Soporte a la Investigación Experimental (SCSIE), Universitat de València, for ProteomeXchangesubmission. Dr. Andrea Peralta for assisting with the use of the ultracentrifuge at Instituto de Biotecnología, Centro de Investigación en Ciencias Veterinarias y Agronómicas (CICVyA) Instituto Nacional de Tecnología Agropecuaria (INTA), Argentina and Dra. Paula Pérez at INBIRS, UBA-CONICET, Facultad de Medicina, Universidad de Buenos Aires.

## Conflict of interest

The authors declare that the research was conducted in the absence of any commercial or financial relationships that could be construed as a potential conflict of interest.

## Publisher’s note

All claims expressed in this article are solely those of the authors and do not necessarily represent those of their affiliated organizations, or those of the publisher, the editors and the reviewers. Any product that may be evaluated in this article, or claim that may be made by its manufacturer, is not guaranteed or endorsed by the publisher.
