# Supplementary figures and images for: Extracellular vesicles from Trypanosoma cruzi-dendritic cell interaction show modulatory properties and confer resistance to lethal infection as a cell-free based therapy strategy
Source: Front Cell Infect Microbiol. 2022 Nov 16;12:980817. doi: 10.3389/fcimb.2022.980817 (PMC9710384; doi:10.3389/fcimb.2022.980817)

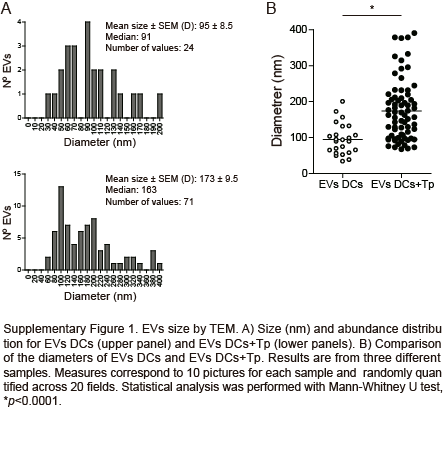

Supplement: Supplementary file 1 [file Image_1.tiff]

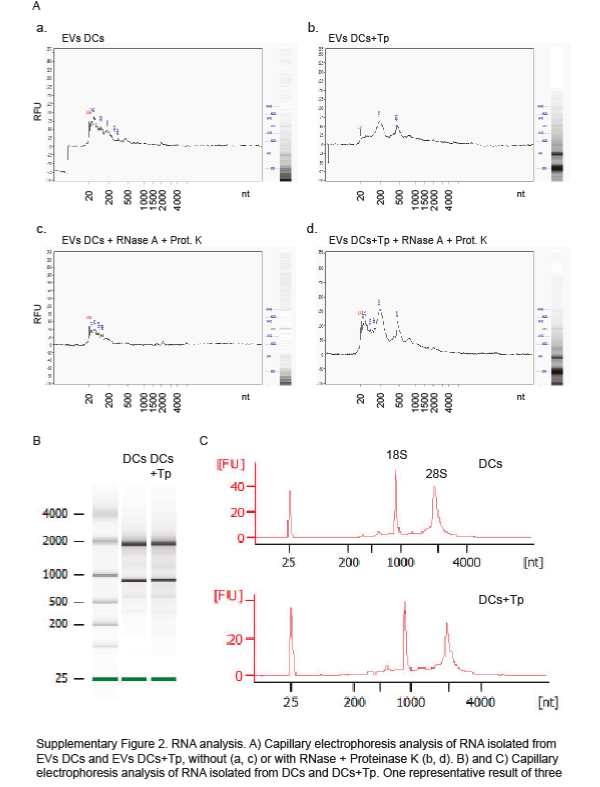

Supplement: Supplementary file 2 [file Image_2.tiff]

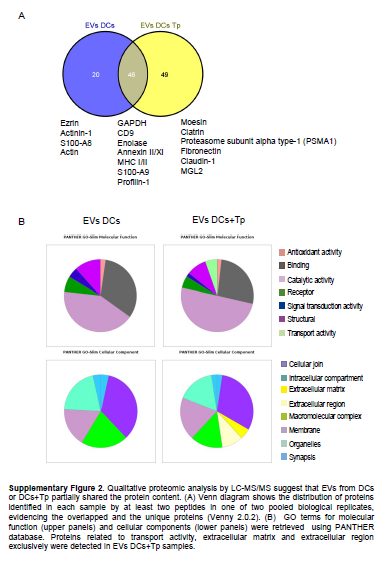

Supplement: Supplementary file 3 [file Image_3.tiff]

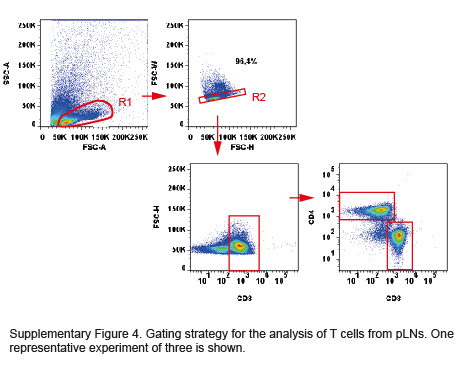

Supplement: Supplementary file 4 [file Image_4.tiff]
